# Supplementary material for: The heme-responsive PrrH sRNA regulates Pseudomonas aeruginosa pyochelin gene expression
Source: mSphere. 2023 Oct 6;8(5):e00392-23. doi: 10.1128/msphere.00392-23 (PMC10597452; doi:10.1128/msphere.00392-23)
Supplement: Supplemental Figures and Tables — Figures S1 to S5 and Tables S1 and S2. [file msphere.00392-23-s0003.pdf]

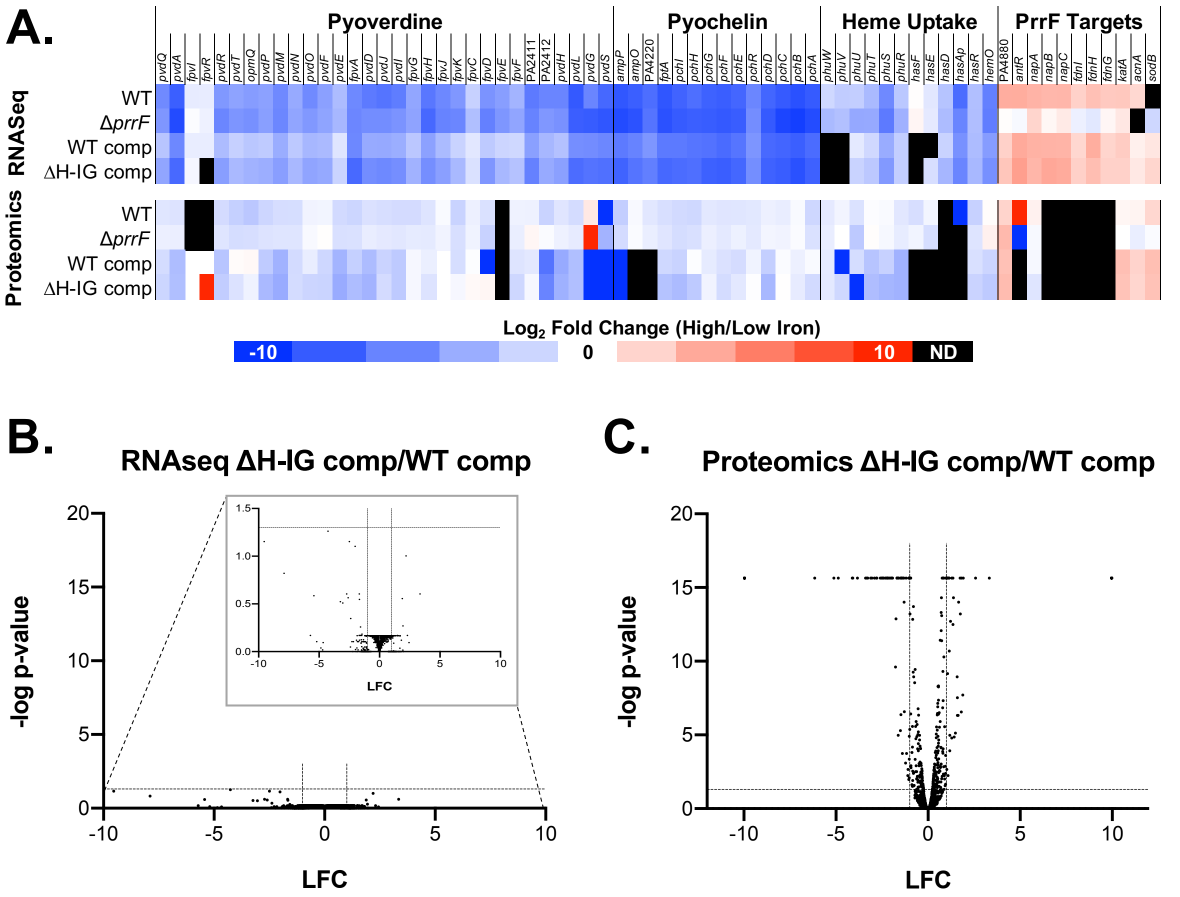

Fig S1. ΔH-IG results in significant changes to the proteome but not the transcriptome. (A) RNAseq and proteomics (E) are displayed as heat maps from an average of 3 or 5 biological replicates (respectively) and shown as the $\log_2$ fold change of the abundance ratio between high iron and low iron. Undetected proteins (ND) are in colored black. For all experiments, samples were collected after 8 hours of aerobic growth in M9 media supplemented with 50 nM $FeCl_3$ (-Fe, low iron), 100 µM $FeCl_3$ (+Fe, high iron), or 5 µM heme at proteomics, samples were collected after 8 hours of aerobic growth in M9 me37˚C. (B-C) Volcano plots from RNAseq (B) and proteomics (C) comparing the ΔH-IG comp to WT comp. For both RNAseq and dia supplemented with 50 nM $FeCl_3$ (-Fe, low iron) or 100 µM $FeCl_3$ (+Fe, high iron) at 37˚C. The $\log_2$ fold change is shown on the x-axis and the -log of the FDR p-value is on the y-axis. Horizontal dashed lines indicate FDR p=0.05 and vertical dashed lines indicate LFC = ±1.

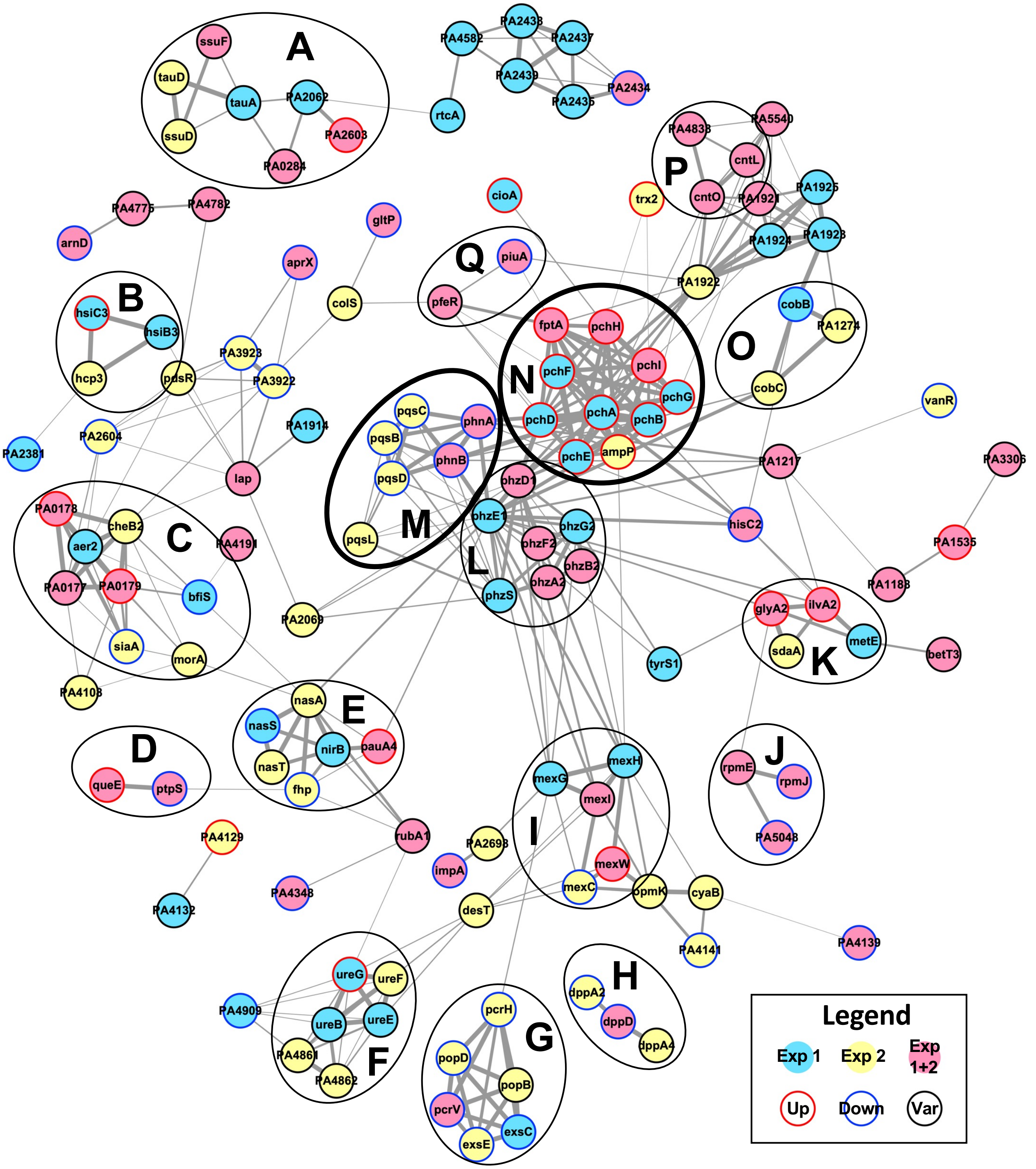

A- Sulfur metabolism
B- H3 Type 6 secretion system
C- Chemotaxis/motility
D- Folate biosynthesis
E- Nitrate assimilation
F- Nitrogen metabolism (urease)
G- Type 3 secretion system
H- Peptide transport
I- Antimicrobial efflux systems
J- Translation
K- Amino acid metabolism and biosynthesis
L- Phenazine biosynthesis
M- Alkylquinolone biosynthesis
N- Pyochelin
O- Cobalamin biosynthesis
P- Zinc uptake
Q- Iron acquisition

Fig S2. STRING network of dysregulated proteins.

A **Sulfur metabolism**

PA0284	hypothetical protein
PA2062	probable pyridoxal-phosphate dependent enzyme
PA2603	probable thiosulfate sulfurtransferase
PA3441	probable molybdopterin-binding protein SsuF
PA3444	SsuD
PA3935	taurine dioxygenase TauD
PA3938	probable periplasmic taurine-binding protein precursor TauA

B **H3 T6SS**

PA2365	HsiB3
PA2366	HsiC3
PA2367	Hcp3

C **Chemotaxis/motility**

PA0172	SiaA
PA0173	chemotaxis specific methylesterase CheB2
PA0176	aerotaxis transducer Aer2
PA0177	probable purine-binding chemotaxis protein
PA0178	probable two-component sensor
PA0179	probable two-component response regulator
PA4197	BfiS
PA4601	motility regulator MorA

D **Folate biosynthesis**

PA0975	probable radical activating enzyme QueE
PA2666	probable 6-pyruvoyl tetrahydrobiopterin synthase, ptpS

E **Nitrate assimilation**

PA1779	assimilatory nitrate reductase NasA
PA1781	assimilatory nitrite reductase large subunit NirB
PA1785	NasT
PA1786	NasS
PA2040	Glutamylpolyamine synthetase PauA4
PA2664	flavohemoprotein Fhp

F **Nitrogen metabolism (urease)**

PA4891	urease accessory protein UreE
PA4892	urease accessory protein UreF
PA4893	urease accessory protein UreG
PA4861	probable ATP-binding component of ABC transporter
PA4862	probable ATP-binding component of ABC transporter
PA4867	urease beta subunit UreB

G **T3SS**

PA1706	type III secretion protein PcrV
PA1707	regulatory protein PcrH
PA1708	translocator protein PopB
PA1709	Translocator outer membrane protein PopD precursor
PA1710	ExsC, exoenzyme S synthesis protein C precursor
PA1711	ExsE

H **Peptide transport**

PA4497	probable binding protein component of ABC transporter DppA2
PA4502	probable binding protein component of ABC transporter DppA4
PA4505	dipeptide ABC transporter ATP-binding protein DppD

I **Antimicrobial efflux systems**

PA4205	MexG
PA4206	MexH
PA4207	MexI
PA4375	MexW
PA4599	MexC

J **Translation**

PA4242	50S ribosomal protein L36 RpmJ
PA5048	probable nuclease
PA5049	50S ribosomal protein L31 RpmE

K **Amino acid metabolism and biosynthesis**

PA1326	threonine dehydratase, biosynthetic IlvA2
PA1927	MetE
PA2443	L-serine dehydratase SdaA
PA2444	serine hydroxymethyltransferase GlyA2

L **Phenazine biosynthesis**

PA1899	probable phenazine biosynthesis protein, PhzA2
PA1900	probable phenazine biosynthesis protein, PhzB2
PA1904	probable phenazine biosynthesis protein, PhZF2
PA1905	probable pyridoxamine 5'-phosphate oxidase, PhzG2
PA4213	phenazine biosynthesis protein PhzD
PA4214	phenazine biosynthesis protein PhzE
PA4217	flavin-containing monooxygenase PhzS

M **Alkylquinolone biosynthesis**

PA0997	PqsB
PA0998	PqsC
PA0999	PqsD
PA1001	PhnA
PA1002	PhnB
PA4190	PqsL

N **Pyochelin**

PA4218	AmpP
PA4221	FptA
PA4222	PchI
PA4223	PchH
PA4224	PchG
PA4225	PchF
PA4226	PchE
PA4228	PchD
PA4230	PchB
PA4231	PchA

O **Cobalamin biosynthesis**

PA1273	cobyrinic acid a,c-diamide synthase CobB
PA1274	conserved hypothetical protein
PA1276	cobalamin biosynthetic protein CobC

P **Zinc uptake**

PA4836	CntL
PA4837	outer membrane protein precursor CntO
PA4838	hypothetical protein

Q **Iron acquisition**

PA2686	two-component response regulator PfeR
PA4514	probable outer membrane receptor for iron transport PiuA

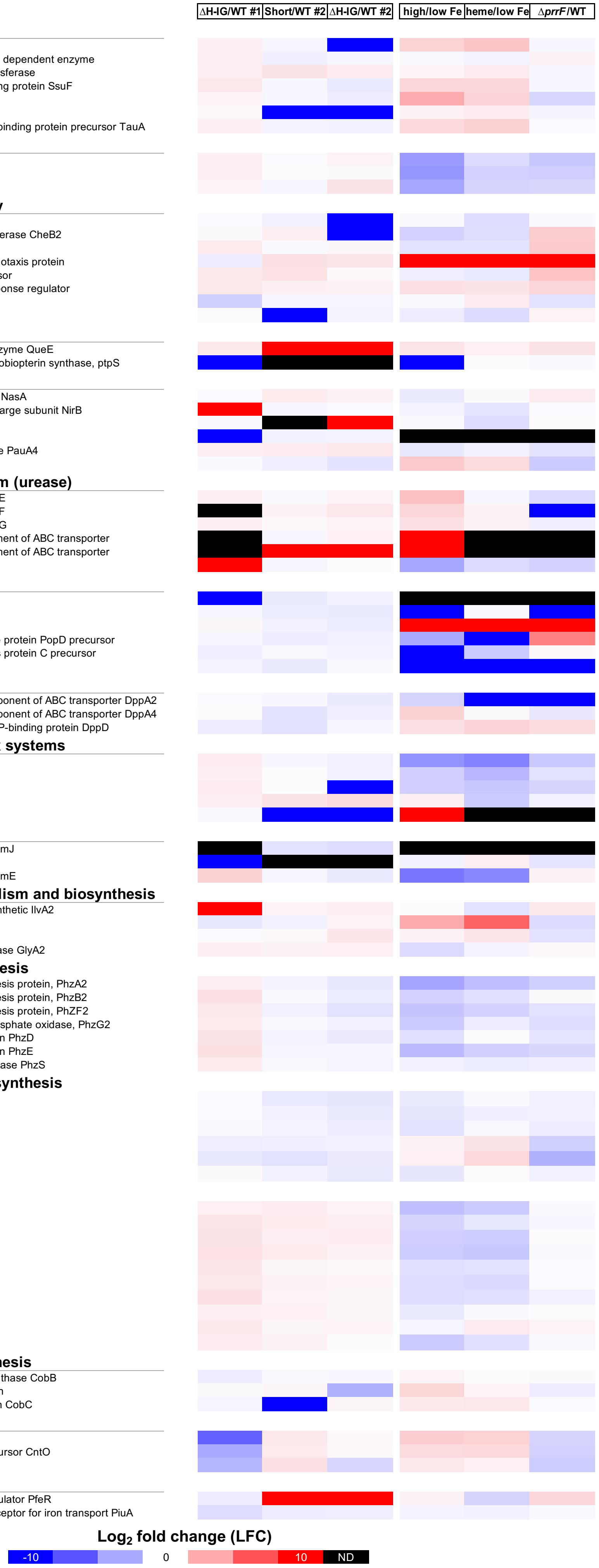

Fig S3. Heatmap of STRING network groups. Proteomics results of proteins identified through STRING network analysis displayed as heatmaps using LFCs calculated from an average of 3 biological replicates. Samples were collected after 8 hours of aerobic growth in M9 media supplemented with 50 nM $FeCl_3$ (-Fe, low iron) or 100 µM $FeCl_3$ (+Fe, high iron), or 5 µM heme at 37˚C. * denotes an FDR p-value <0.05 and ** denotes an FDR p-value <0.005.

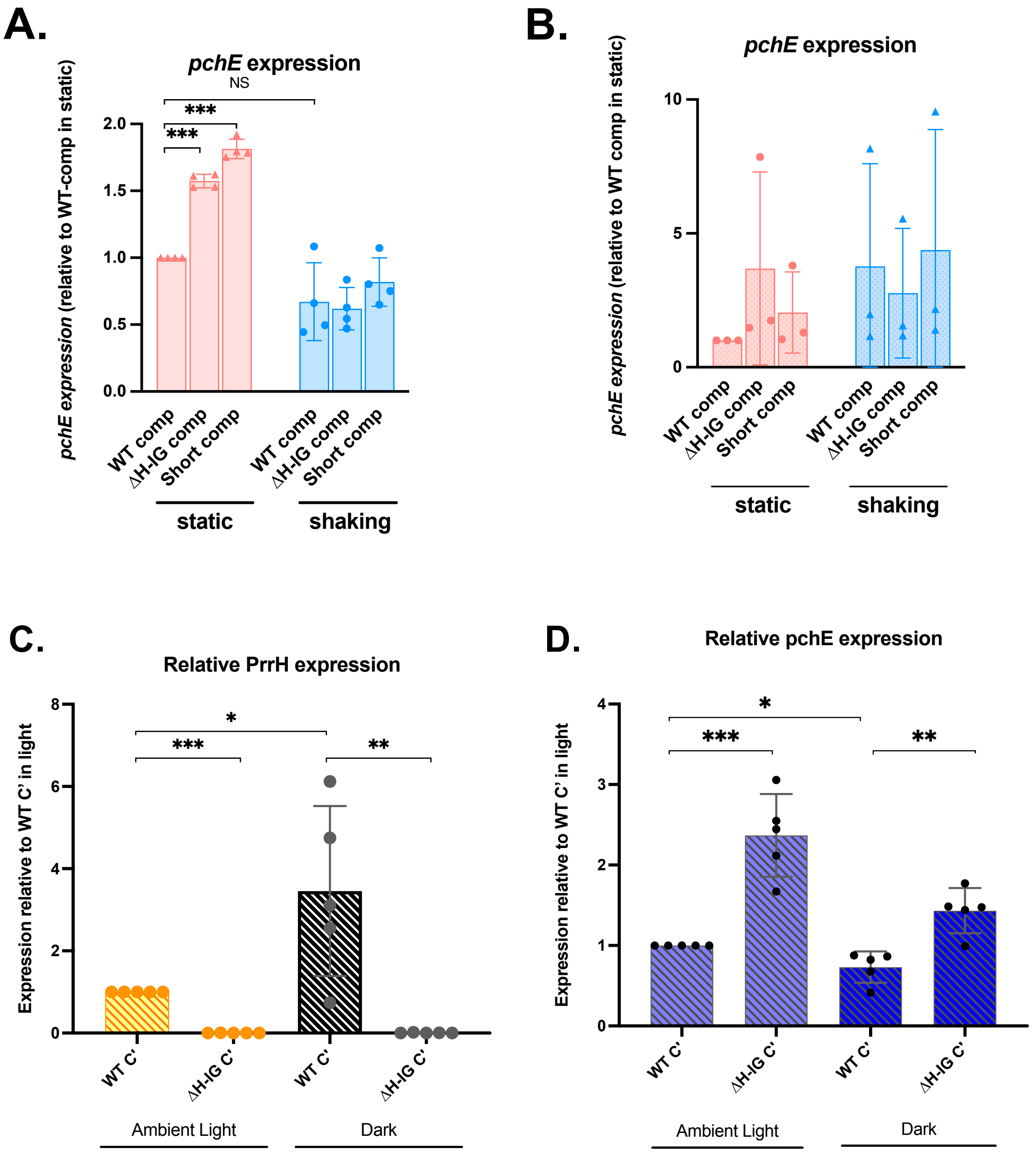

Fig S4. Independent experimental analysis of the effects of static growth and light on PrrH and *pchE.* (A-B). Two independent experiments were used to examine the impact of static growth on PrrH-repression of pchEF. (C-D) Second independent experiment analyzing the impact of light on PrrH-mediated repression of pchE. Data are the average of either 3 (A-B) or 4 (C) or 5 (D) biological replicates in each experiment. The indicated strains were grown in M9 media supplemented with 50 nM $FeCl_3$ grown statically at 37˚C for 8 hours. Significance was calculated using an unpaired *t* test with two-tailed P values where * indicates P<0.05, ** P<0.005, and *** P<0.0005.

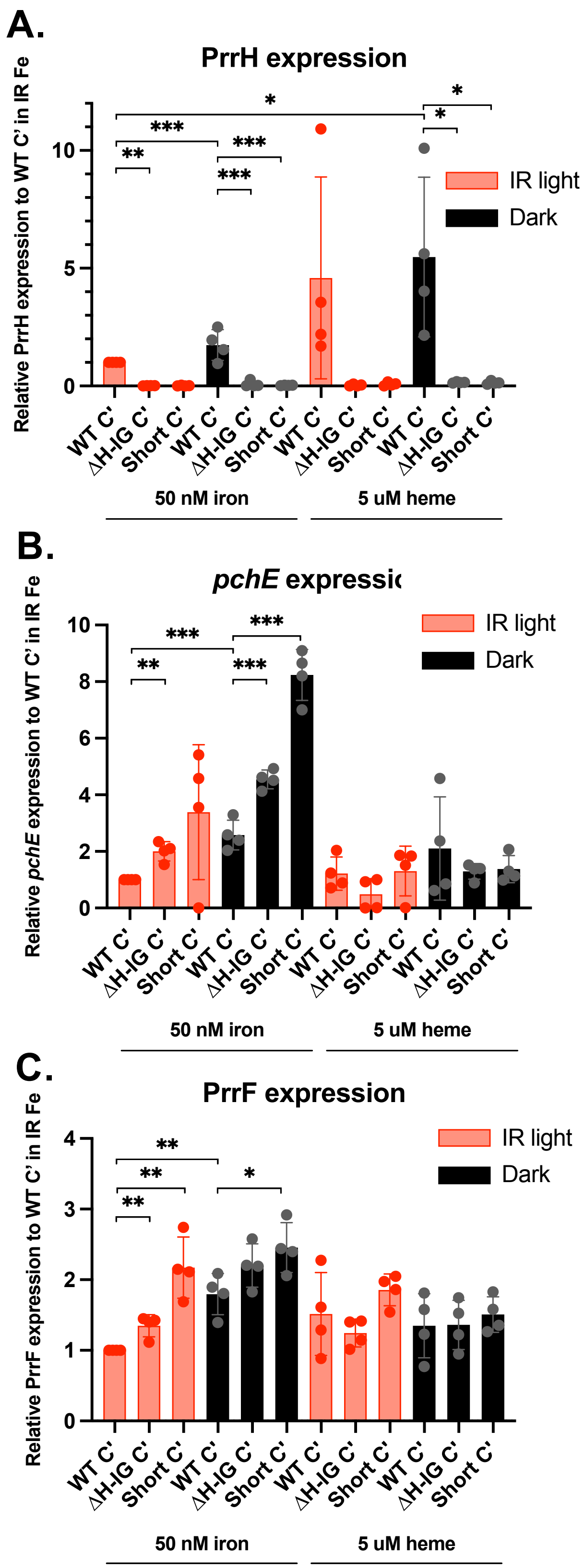

Fig S5. Relative PrrH (A), *pchE* (B), and PrrF (C) transcription levels relative to WT-comp in infrared (IR) light 50 nM $FeCl_3$ condition. Data are an average of 4 biological replicates in M9 media supplemented with 50 nM $FeCl_3$ or 5 μM heme grown statically at 37˚C for 8 hours. Significance was calculated using an unpaired *t* test with two-tailed P values where * indicates P<0.05, ** P<0.005, and *** P<0.0005.

Table 1. Bacterial strains used in this study

Strain	Description	Reference
P. aeruginosa PAO1	Human wound isolated originally isolated in Australia	(10)
PAO1 Δ*prrF*	Mutant with a deletion of the entire *prrF* locus	(11)
PAO1 pUCP18	PAO1 carrying the pUCP18 empty vector	(5)
PAO1 Δ*prrF* pUCP18	PAO1 *prrF* deletion mutant carrying the pUCP18 empty vector	(5)
PAO1 Δ*prrF* WT complement (WT Comp)	PAO1 Δ*prrF* carrying the entire *prrF* locus, including 235 bp upstream of the *prrF1* start site, on the plasmid pUCP18	(5)
PAO1 Δ*prrF* ΔH-IG complement (ΔH-IG Comp)	PAO1 Δ*prrF* carrying the *prrF* locus with a deletion of the PrrH intergenic region on the plasmid pUCP18	(5)
PAO1 Δ*prrF* Short complement (Short Comp)	PAO1 Δ*prrF* carrying the entire *prrF* locus, including 184 bp upstream of the *prrF1* start site, on the plasmid pUCP18	(5)
PAO1 Δ*pchEF*	PAO1 with a chromosomally deleted *pchEF*	(12)

Table 2. Primers and probes used in this study

Primer/probe	Sequence (5’- 3’)
PrrF1 northern blot probe	CGT GGG TCT CTC AGC TTA CCG GCT GAT CT
PrrF2 northern blot probe	CTT GGT CTC TCA GCT TAC CTG CTG GCC T
PrrH northern blot probe	GTT CTC ATT CAT TCC ATC GCC AGC CGA
PrrF qRT-PCR forward primer	AAC TGG TCG CGA GAT CAG C
PrrF qRT-PCR reverse primer	CCG TGA TTA GCC TGA TGA GGA G
PrrF qRT-PCR probe	CCC ACG CAG TCG GAC TCT TCA GAT T
PrrH qRT-PCR forward primer	ATT CGG CCG GAG ACG ACC GTT
PrrH qRT-PCR reverse primer	CTG GCG ATG GAA TGA ATG AGA ACC G
PrrH qRT-PCR probe	CGA CCA GTT GGT GTA ATA ATA ACT ATT
pchE qRT-PCR forward primer	CGG CGA TCA ATA CCA TCG AC
pchE qRT-PCR reverse primer	AAG ACC GAC AGA TCG AAG TCC A
pchE qRT-PCR probe	TGA ACG CAT CGG ATC GCT TGC TG
